# Not Just Anticoagulation—New and Old Applications of Heparin

**DOI:** 10.3390/molecules27206968

**Published:** 2022-10-17

**Authors:** Lixuan Zang, Haomiao Zhu, Kun Wang, Yonghui Liu, Fan Yu, Wei Zhao

**Affiliations:** 1 State Key Laboratory of Medicinal Chemical Biology, College of Life Sciences, College of Pharmacy, Nankai University, 38 Tongyan Road, Jinnan District, Tianjin 300350, China; 2 National Glycoengineering Research Center and Shandong Key Laboratory of Carbohydrate Chemistry and Glycobiology, Shandong University, Qingdao 266237, China; 3 Department of Pharmacy, Qilu Hospital, Shandong University, 107 Cultural West Road, Jinan 250012, China; 4 School of Chemistry, Tiangong University, Tianjin 300387, China

**Keywords:** heparin, applications of heparin, challenges of heparin therapy

## Abstract

In recent decades, heparin, as the most important anticoagulant drug, has been widely used in clinical settings to prevent and treat thrombosis in a variety of diseases. However, with in-depth research, the therapeutic potential of heparin is being explored beyond anticoagulation. To date, heparin and its derivatives have been tested in the protection against and repair of inflammatory, antitumor, and cardiovascular diseases. It has also been explored as an antiangiogenic, preventive, and antiviral agent for atherosclerosis. This review focused on the new and old applications of heparin and discussed the potential mechanisms explaining the biological diversity of heparin.

## 1. Introduction

A sulfated glycosaminoglycan, heparin was named after its initial isolation from liver tissue a century ago [1,2]. It exists in the lung, vascular wall, intestinal mucosa, and so on. Due to its unique pentasaccharide sequence, heparin exerts anticoagulant activity after binding with antithrombin, inhibits the activation of factors Xa and IIa in the coagulation cascade, and finally exerts anticoagulant activity. Heparin is a natural anticoagulant in animals and is the most widely used anticoagulant treatment [3,4]. Heparin is usually used to prevent or treat thrombosis-related diseases, such as embolic diseases and myocardial infarction, or for blood anticoagulation during surgeries such as cardiovascular surgery, cardiac catheterization, cardiopulmonary bypass, and hemodialysis. In recent years, with the development of synthetic low-molecular-weight heparin (LMWH) and heparin derivatives, these compounds have been proven through various activity studies to have many pharmacological effects, such as anti-inflammatory, antiangiogenic, antitumor, and antimetastatic effects, in addition to anticoagulant effects [5,6,7]. At the same time, as a natural water-soluble polysaccharide, heparin has good biocompatibility, so there have also been studies on combining heparin with nanomaterials to give it wider medical application value.

**Figure 1:**
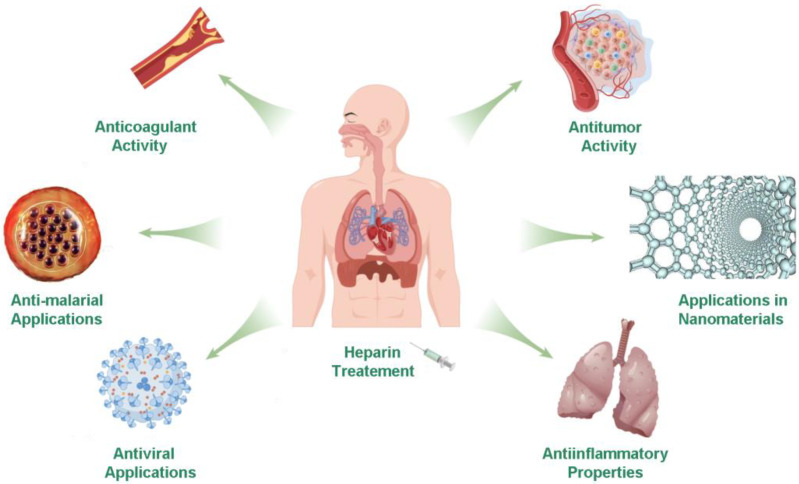
The applications of heparin.

In particular, the infection caused by severe acute respiratory syndrome coronavirus 2 (SARS-CoV-2, COVID-19) spread rapidly all over the world in 2019. Patients with severe COVID-19 may eventually develop systemic thrombovasculitis, leading to severe organ dysfunction [8]. Heparin can reduce the systemic symptoms of patients, which indicates that heparin may have an undeveloped effect in the treatment of COVID-19 [9,10]. Therefore, in this review, we briefly summarized the new and old applications and related mechanisms of heparin and its derivatives (Figure 1).

## 2. The Structure of Heparin

Heparin belongs to the glycosaminoglycans (GAGs), which are composed of a linear set of negatively charged polysaccharides. According to differences in the polysaccharide unit, GAGs include heparin sulfate, chondroitin sulfate, hyaluronic acid, dermal sulfate, keratin sulfate, etc. There are three common and commercially available anticoagulant heparins: unfractionated heparin (UFH), low-molecular-weight heparin (LMWH), and ultralow-molecular-weight heparin (ULMWH). UFH is a natural heparin extracted from animal tissues, with a molecular weight of 3000 to 30,000 Da [7,11,12]. The heparin commonly used in treatment is mainly composed of the trisulfated disaccharide L-iduronic acid-2-sulfate and D-glucosamine-N,6-disulfate. Moreover, it is worth noting that these regular sequences are interrupted by undersulfated (occasionally persulfated) sequences containing D-glucosamine acid and N-acetylated D-glucosamine.

Various enzymes are involved in the biosynthetic pathway of heparin, such as synthetase, epimerase, etc. Moreover, heparin is further modified in vivo by N-deacetylation/N-sulfation of the glucosamine units, C-5 epimerization of the glucuronic acid, and O-sulfation at different sites of the chain [13,14]. These modifications lead to the heterogenization of heparin structure, and realize the regulation of multiple cellular mechanisms in organisms.

About 70% of the heparin polymer is highly sulfated and consists of repeated trisulfated disaccharide units [15]. The remainder is low-sulfated heparin, in which some sites are desulfated, such as 6-O-desulfated glucosamine, N-acetylglucosamine instead of N-sulfated glucosamine, and glucuronic acid instead of iduronic acid. Different extraction sources produce different types of heparin, for example, in the heparin from bovine lung, the content of N-acetyl glucosamine is lower and sulfation is higher [16]. However, there also exist conserved sequence forms among different type of heparins. Around one third of UFH has a specific five-sugar sequence of which the central glycogen is 3-O-sulfated glucosamine. This sequence realizes anticoagulant activity by binding antithrombin (AT) [17,18].

As part of the rapid development of research related to heparin, especially the study of the molecular mechanism of anticoagulation, the LMWHs, which are produced by incomplete depolymerization of UFH by chemical or enzymatic methods, have become a new research field [19]. LMWH is more uniform, with a chain between 2000 and 8000 Da (average 4500 Da) [20]. In addition, the pharmacokinetics and anticoagulant effects of LMWHs are different, as the depolymerization method is different. With this characteristic, the administration of LMWHs could be personalized according to individual patients in the prevention and treatment of venous thrombosis and pulmonary embolism, making LMWHs the first choice for many indications [21,22,23]. Moreover, LMWHs not only play an important role in thrombus treatment, but can also be used in a variety of other indications, such as maintaining vascular patency during hemodialysis and arterial bypass grafting [24], and the prevention of acute bronchial asthma contractions [25].

## 3. Anticoagulant Activity

**Figure 2:**
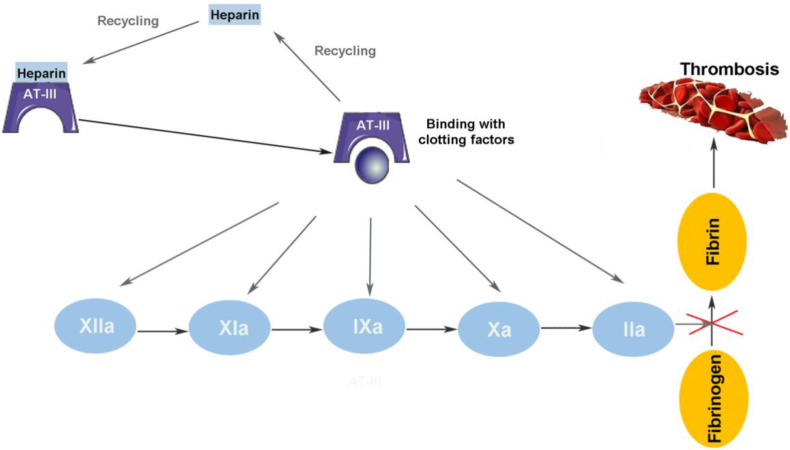
The interactions of heparin with the anticoagulant systems.

Coagulation is an extremely complex process in organisms. When a series of coagulation factors are continuously activated in a specific order and finally form insoluble polymers, a coagulation reaction occurs. As one of the most widely used anticoagulants for the prevention and treatment of thromboembolic diseases, the anticoagulant effect of heparin is mainly mediated by antithrombin III (AT-III) [26]. Heparin combines with AT-III lysine residues to form a reversible complex, which changes the configuration of AT-III, fully exposes the active site of arginine, and quickly combines with the serine active centers of factor IIa (thrombin) and IXa, Xa, Xia, and XIIa to accelerate the inactivation of coagulation factors, effectively preventing the formation of blood clots and playing an anticoagulant role. When inactivating IXa/IIa, heparin must combine with AT-III and coagulation factor to form a ternary complex, while when inactivating Xa, it only needs to combine with AT-III [27,28,29]. Once the heparin–AT-III coagulation factor complex is formed, heparin can be dissociated from the complex and reused (Figure 2). To form the heparin–AT-III thrombin ternary complex, the chain length of the heparin molecule needs at least 18 monosaccharide units [30].

**Figure 3:**
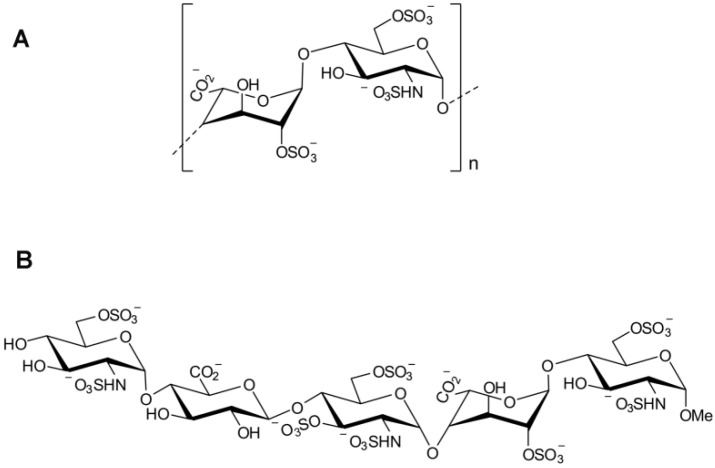
(**A**) The repeated sulfate disaccharides structure of heparin. (**B**) The pentasaccharide responsible for the anticoagulant activity of fondaparin.

Heparin plays an important role in the treatment and prevention of venous thrombosis (VTE). When it occurs as hypercoagulation in pregnancy, VTE can lead to higher maternal morbidity. Heparin is safer than other anticoagulants when used during pregnancy [31,32,33,34,35]. Due to their animal origin and biosynthesis, GAGs have highly variable chain and sulfation patterns, which ultimately prevent the perfect purification of UFH. This became fatal during the “heparin contamination crisis” of 2007 and 2008 [36]. LMWH was developed in the 1980s and is produced by chemical or enzymatic degradation of heparin. The carbohydrate chain contains an average of 15 monosaccharide units and has a molecular weight of 3–8 kDa. The purpose of splitting heparin into LMWH is to reduce the length of the glycosaminoglycan chain to make these preparations easier to absorb, especially when delivered through subcutaneous injection. LMWH exhibits better subcutaneous bioavailability and a longer half-life (3–6 h) due to its low affinity for plasma proteins, endothelial cells, and blood cells; it can even be used once or twice daily without laboratory monitoring. Since the 1990s, LMWH has been recommended for the prevention and treatment of thromboembolic events because of its association with fewer adverse events than UFH [37,38]. At present, the common commercial ULMWH is fondaparin, which is a chemically synthesized pentosan methyl derivative (active fragment of UFC and LMWH) with a molecular weight of 1728 Da [39,40]. Due to its production characteristics, the product of fondaparin has stable properties, uniform structure, and easy derivation and modification of functional groups. It is the focus of heparin drug research at present (Figure 3).

## 4. Antitumor Activity

As early as the 20th century, there were reports on the use of heparin in antitumor research. First, because the blood of patients with advanced cancer is usually in a hypercoagulable state, it is necessary to use heparin for anticoagulant treatment while fighting cancer. Several clinical reports have observed that the use of heparin or heparin derivatives in the treatment of cancer-related thromboembolic diseases seems to prolong the survival time of cancer patients, which has aroused interest in the antimetastatic properties of heparin [41,42]. This characteristic seems to be related to a variety of potential anticoagulant and nonanticoagulant mechanisms.

Cancer metastasis is closely related to angiogenesis and cell adhesion. Some authors believe that LMWH and recombinant tissue factor pathway inhibitors (TFPIs) prevent angiogenesis induced by various angiogenesis factors (such as VEGF). These findings suggest that the interaction between LMWH and TFPI plays a key role in the regulation of angiogenesis. Meanwhile, Mousa and Norrby et al. also proved the potential of LMWH for angiogenesis of new tumors using a chorioallantoic membrane assay (CAM) angiogenesis model [43,44]. The regulation of the interactions between chemokines and their receptors constitutes another effect of heparin on cancer metastasis. Among these, the interaction between chemokine CXCL12 and its receptor CXCR4 is the basis of the metastasis of breast cancer. LMWH inhibited the interaction between CXCL12 and CXCR4, thereby reducing the metastatic spread of breast cancer cells in mice [45]. In animal cancer models, UFH also reduced tumor cell adhesion and LMWH reduced metastatic burden and primary tumor growth, but LMWH did not increase overall survival in patients with solid tumors [46,47]. Galectin 3 is a beta-galactoside-binding protein that is commonly overexpressed in most types of cancer. It has also recently been shown that it can be inhibited by heparin, thereby inhibiting tumor cell metastasis. Although the mechanism of this inhibition of metastasis is still unclear, the linkage of heparin has a significant inhibitory effect on galectin-3-mediated cancer cell metastasis [48].

In addition, heparin can also help to inhibit the proliferation of several cell types. It mainly exerts this antiproliferative effect by modifying the protein kinase C dependent signal transduction pathway and inhibiting some protooncogenes such as c-myc and c-fos. Heparin has been shown to inhibit the phosphorylation (i.e., activation) of MAPK as part of the protein kinase C signaling cascade [49]. Heparin can also affect invasive cancer metastasis by inhibiting the activity of heparanase, which affects the integrity of the extracellular matrix (ECM) and the basement membrane of the vascular wall [50,51]. At present, clinical results show that the inhibition by heparin and LMWH of tumor tissue-related clots is conducive to the better efficacy of radiotherapy and chemotherapy drugs [52,53,54]. The current clinical guidelines state that LMWH is the first choice for antithrombotic therapy in cancer patients.

## 5. Anti-Inflammatory Properties

Inflammation is a series of defensive responses to harmful stimuli, often involving the local vascular system and immune system. Since endogenous heparin is only stored in mast cell granules, it is not surprising that drug-grade heparin has immunomodulatory properties. Filkin et al. first studied the protective effect of heparin on lipopolysaccharide (LPS)-induced shock in 1968 and effectively reduced the mortality of LPS model mice [55]. However, apart from the high-affinity binding of antithrombin to heparin through a unique pentasaccharide sequence [56], there is no conclusive evidence describing other examples of “specific binding”. Therefore, the mechanism of the anti-inflammatory effect of heparin is complex and not fully understood. It is known that heparin exerts anti-inflammatory effects through a variety of mechanisms [57]. Heparin can not only inhibit the specific functions of neutrophils [58,59] and reduce the migration of eosinophils and vascular permeability, thereby alleviating inflammatory reactions, but also interact with cytokines such as NF-κB, NF-α, IL-6, IL-8, and IL-1β in the vascular endothelium [60,61], preventing activation of the innate immune system. In addition, heparin can inhibit the proliferation of vascular smooth muscle cells [57]. The anti-inflammatory effect of heparin is regulated by many factors, such as source, length, and structure, which cause changes in the anti-inflammatory effect [11].

At present, some studies suggest that heparin can combine with cytokines, chemokines, and acute-phase proteins, including IL-8, platelet growth factor 4 (PGF4), matrix-derived factor 1a, neutrophil elastase, and P- and L-selectin, to exert anti-inflammatory effects [31,62,63].

Several recent studies have shown that heparin and its derivatives have good anti-inflammatory effects in asthma, chronic obstructive pulmonary disease (COPD), acute lung injury, and sepsis [64]. In particular, the good therapeutic effect of nonanticoagulant heparin on sepsis is very exciting. Sepsis is a life-threatening organ dysfunction caused by an imbalance in the body’s response to infection. It has become one of the most important causes of death in patients with clinically critical illnesses, with the “three high characteristics” of high prevalence, high mortality, and high treatment costs. Histone is one of the regulatory mediators in sepsis. As a negatively charged, highly sulfated polysaccharide structure, heparin can hinder the interaction between positively charged histones and platelets and may represent a potential solution for sepsis through regulation of the inflammatory response [65]. In addition, through the glycocalyx, heparin can also participate in the cross-endothelial channels of leukocytes and the endothelial and cross-endothelial effects of inflammatory cytokines [66], inhibit the inflammatory response, repair damaged endothelial cells, and rebuild vascular barrier function. It is well known that the surface of endothelial cells is covered by a macromolecular reticular structure called the glycocalyx, which is a glycoprotein with syndecan-1 as the core [67]. In recent years, some studies have shown that UFH, as a HS analogue, can participate in the mobilization of glycocalyx core protein syndecan-1 to reconstruct the glycocalyx left on the cell surface and protect it from shedding, so as to achieve the integrity of the cell surface and ensure a good vascular barrier [68,69].

Hala et al. studied the preparation and characterization of a polyelectrolyte multilayer (PEM) coating which combined the anti-inflammatory activity of heparin as a polyanion and the potential release of naproxen. PEM containing heparin was shown to reduce cell adhesion and IL-β as a substitute for polyanions to form multilayers [70].

## 6. Antiviral Application

Research on heparin as an antiviral treatment has surged again following the recent outbreak of COVID-19. Amongst the long history of heparin, there were early studies on the inhibition of herpes simplex virus by heparin in vitro as early as the mid-20th century [71]. Heparin inhibits herpes simplex virus as a natural inhibitor with a unique sulfonation mode [72,73]. Subsequently, there were studies on the inhibitory potential of heparin on a variety of RNA and DNA viruses, such as HIV, in vitro [74,75,76].

Heparan sulfate (HS) on the cell surface can be used as a coreceptor by viruses, helping many viruses attach to or enter target cells. Modhiran et al. showed that a heparan sulfate analogue (PG545) can be used as an inhibitor of virus–cell adhesion, which can effectively prevent the transmission of dengue virus in clinical settings [77,78]. Similarly, by affecting cell adhesion, HS promotes the adhesion and entry of rabies virus (RABV) into target cells [79]. It should be noted that heparin is not necessary for cell adhesion of Zika virus, but it participates in virus replication and induces apoptosis of infected cells [80].

In recent years, due to the frequent outbreaks of severe acute respiratory syndrome (SARS), studies on the effects of heparin and its analogues as antiviral treatments have been ongoing. It was recently shown in an in vitro study that SARS-CoV-2 first attaches to heparan sulfate proteoglycans before interacting with ACE2 [81]. Similarly, the study of Clausen et al. [82] also showed that the spike protein of SARS-CoV-2 can bind to the cell surface through both HS and ACE2 protein receptors. All of the abovementioned results indicate that HS is required for the attachment of SARS-CoV-2 to the cell surface. Therefore, it is now generally believed that in severe acute respiratory syndrome (SARS)-coronavirus (COV)-2 infection, heparin can be used as a bait receptor to bind the SARS-CoV-2 spike protein, inhibiting the binding of the virus to HS and reducing the infectivity of the virus [83,84]. In addition, in patients with severe COVID-19 infection, platelet activation, the increase in blood viscosity caused by high fibrinogen levels, and the increase in heparanase expression [85] all further increase the risk of vascular disease [86]. Although previous anticoagulant treatments have failed in critical illnesses, convincing observations of coagulation dysfunction and high venous thromboembolism rates in COVID-19 increase the possibility that heparin may benefit the prognosis of patients. Moreover, heparin has beneficial effects on inflammation, which is associated with COVID-19. Heparin binds and regulates the activity of many inflammatory proteins, including IL-8, platelet growth factor 4, neutrophils, elastase control, CD11b/CD18, etc. [85]. In a randomized clinical trial by Alex et al., therapeutic-dose LMWH reduced major thromboembolism and death compared with institutional standard heparin thromboprophylaxis among inpatients with COVID-19 with very elevated D-dimer levels [87].

According to the study of Hidesaku et al., COVID-19 shows some characteristics similar to disseminated intravascular coagulation (DIC). Nafamostat mesylate (NM) is a drug used for treating DIC, and is also expected to be a drug used for treating COVID-19. However, the anticoagulant effect of NM is weak. Heparin can make up for this shortcoming and can be used in combination to treat COVID-19. Therefore, heparin as a combined drug in COVID-19 may be a future research direction [88].

## 7. Application of Heparin in Malaria

Malaria is caused by infected with Plasmodium, which is an infectious disease. Malaria can be spread by mosquito bites and then propagate in the liver of patients, leading to infection, causing the destruction of red blood cells, and then continuing to reproduce and be destroyed.

At present, with global warming, globalization, and the living environment continuing to deteriorate in some areas due to war, the incidence rate of malaria is continuing to increase. Although it has attracted attention globally, because the existing first-line drugs cannot effectively treat malaria and there has been no breakthrough in vaccine research, there is an urgent need for new treatment schemes and new mechanisms to improve treatment efficiency.

GAGs can bind to Plasmodium parasite red blood cells (PBRC), thus blocking the binding of PRBC to various host-cell surface receptors [89,90,91,92]. Negatively charged polysaccharides, such as heparin and chondroitin sulfate, have the ability to bind to PBRC, thus achieving antimalaria effects. Xuerong Dong et al. constructed an intraerythrocytic parasite-targeted nanostructured lipid carrier (NLC) that was developed for potentiation of artemether (ARM) by combination with PPIX and iron-loaded transferrin (holo-Tf). ARM and PPIX were co-loaded into NLCs with high entrapment efficiency. A targeting ligand heparin (HP) was then electrostatically adsorbed onto the periphery of the NLCs, followed by conjugation with holo-Tf to obtain the final formulation, Tf-HP-NLC/ARM/PPIX. This delivery system showed increased inhibitory activity against Plasmodium falciparum in culture [93].

However, heparin has anticoagulant and bleeding properties, so it will increase the risk of infection [94,95,96,97], which is a disadvantage in the treatment of malaria. Heparin has been shown to bind merozoites inside late-stage pRBCs. This finding can be used as a new direction in future research into antimalarial drugs.

## 8. Application of Heparin in Nanomaterials

Heparin nanocomposites first appeared in 2008. The authors of that first study synthesized hollow capsules based on an iron heparin complex and multilayer films, which significantly increased anticoagulation time. The capsules can be used as injectable anticoagulant carriers for parenteral injection to treat iron deficiency [98]. Since then, heparin nanomaterial composites have been continuously explored and innovated in the field of medical applications.

Nanomaterials and heparin are connected by covalent bonds or electrostatic interaction. Heparin, as a natural water-soluble polysaccharide, has good biocompatibility and a variety of biological functions. When combined with nanomaterials, it shows increased stability and water solubility, and can improve the targeting of molecules. Studies have shown that heparin nanoparticles have strong growth-factor-loading capacity and are cytokines that maintain activity for a long time [99,100,101]. Heparin nanoparticles have strong potential value in the field of drug delivery. Heparin nanomaterials also show significant value in the treatment of other diseases. Qi Tan et al. constructed a heparin chitosan nanoparticle-immobilized scaffold, which showed high-efficiency vascular endothelial growth factor (VEGF) localization and release ability in vitro and significantly increased fibroblast infiltration and extracellular matrix generation, and accelerated angiogenesis in the subcutaneous implantation model of mice [102]. Yasutaka et al. constructed LMWH protamine nanoparticles (lmwh-h/P NPs) as a carrier for heparin-binding growth factor. Fibroblast growth factor (FGF-2) combined with nanoparticles significantly extended the biological half-life of FGF-2 [103]. Jeong et al. constructed a PEG-LHT7/TRAIL/Protamine nanocomplex within a PEG-LMWH-taurocholate conjugate (LHT7). The selective cytotoxicity of tumor necrosis factor-related apoptosis-inducing ligand (TRAIL) to cancer cells, but not to normal cells, makes it an attractive candidate for cancer therapeutics. However, the disadvantages of TRAIL, such as physicochemical instability and short half-life, limit its further clinical applications. This heparin nanoparticle improved the short life of this drug and not only had anti-angiogenesis effect in the treatment of tumors, but also uniformly induced tumor cell apoptosis [104].

In conclusion, heparin nanocomposites, with their superior pharmacokinetic properties and biological functions, have prompted scientists to explore new drug delivery systems for improved therapeutic effects. Combining the advantages of the two can also overcome the inherent limitations of the structures of heparin and nanomaterials.

## 9. Challenges of Heparin Therapy

The effects of heparin are not exclusively positive. Some adverse reactions reported during heparin treatment are closely related to the biological activity of heparin itself. Firstly, among its own characteristics, heparin has a short half-life which requires frequent administration in clinical applications, leading to poor compliance of patients. At present, attempts are being made to extend the half-life using slow-release preparations [105].

Heparin is the most effective anticoagulant in clinical use, but it also carries a certain risk of bleeding [106,107]. Especially in elderly individuals or those with renal insufficiency, this needs special attention. Bleeding-related complications from heparin can range in severity from mild symptoms such as injection site hematomas to potentially fatal events such as intracranial hemorrhage. Some researchers have also observed in clinical settings that hematoma at the puncture site can occur when heparin is used for epidural anesthesia or spinal cord puncture; this symptom is indeed extremely dangerous because it carries the risk of causing paralysis. In addition, with increasing chain length, heparin has enhanced binding ability with positively charged molecules such as platelet 4 (PF4), which can form new antigen complexes to destroy receptors on platelets and endothelial cells, leading to thrombocytopenia (HIT). HIT can also cause skin damage or even necrosis at the heparin injection site. Moreover, HIT is a very noteworthy problem because of the ease of relapse [108]; immune memory is cleared several months after the onset of the disease. After the reintroduction of heparin, the risk of HIT recurrence is the same as that of patients not affected by heparin. In addition, some studies have shown that heparin can bind to bone protein, affect osteoblast bone synthesis, increase osteoblast absorption, and finally reduce bone mass, even leading to osteoporosis [109]. This is also one of the common side effects of long-term use of heparin and LMWH [110,111]. Although some studies have found that short-term use of LWMH (3–6 months) does not affect bone mineral density, this should be noted in pregnant women, the elderly, and children as the effect is long-lasting and irreversible [112].

Increases in eosinophils, hyperkalemia, and other side effects are not common and can recover with the cessation of heparin treatment. Some patients with special diseases, such as patients with chronic renal failure, may have adverse reactions such as calcium deposition at the injection site of heparin due to abnormalities in calcium and phosphorus in the body.

## 10. Conclusions

With the discovery of new indications and new possibilities, the role of heparin continues to develop. Although heparin is well known by the public as a mainstream anticoagulant drug, with the deepening of research, heparin and heparin derivatives are showing an edge in various fields. At present, LMWH and UFH are commonly used, but heparin has a complex structure and can be modified, for example, by sulfation and acetylation. At present, the specific efficacy of heparins with different molecular weights has not been studied thoroughly. With the maturing of enzymatic synthesis of sugar chains, high-purity oligoheparin may have broad application prospects. In addition, many proteins can bind to heparin and heparin can produce various pharmacological effects, such as anti-inflammatory, antiviral, antiangiogenic, antitumor, and antimetastatic effects, by interacting with a variety of proteases, protease inhibitors, and chemokines. In this review, we comprehensively discussed the old and new applications of heparin and explained its therapeutic mechanism to a certain extent. Due to the coexistence of clinical benefits and adverse reactions, efforts should be made to optimize the medication regimen or modify the structure–activity relationship to improve the therapeutic effect and reduce the possibility of adverse reactions. At the same time, more clinical studies are necessary in order to provide effective, accurate, and reliable evidence. In short, heparin, as a classic drug that has been used for a century, has gone beyond its traditional role of anticoagulation, and achieved new development potential under the optimization of current research and development.

## Data Availability

Not applicable.
